# scds: computational annotation of doublets in single-cell RNA sequencing data

**DOI:** 10.1093/bioinformatics/btz698

**Published:** 2019-09-10

**Authors:** Abha S Bais, Dennis Kostka

**Affiliations:** Department of Developmental Biology, USA; Department of Developmental Biology, USA; Department of Computational and Systems Biology and Pittsburgh Center for Evolutionary Biology and Medicine, University of Pittsburgh School of Medicine, Pittsburgh, PA 15201, USA

## Abstract

**Motivation:**

Single-cell RNA sequencing (scRNA-seq) technologies enable the study of transcriptional heterogeneity at the resolution of individual cells and have an increasing impact on biomedical research. However, it is known that these methods sometimes wrongly consider two or more cells as single cells, and that a number of so-called doublets is present in the output of such experiments. Treating doublets as single cells in downstream analyses can severely bias a study’s conclusions, and therefore computational strategies for the identification of doublets are needed.

**Results:**

With scds, we propose two new approaches for *in silico* doublet identification: Co-expression based doublet scoring (cxds) and binary classification based doublet scoring (bcds). The co-expression based approach, cxds, utilizes binarized (absence/presence) gene expression data and, employing a binomial model for the co-expression of pairs of genes, yields interpretable doublet annotations. bcds, on the other hand, uses a binary classification approach to discriminate artificial doublets from original data. We apply our methods and existing computational doublet identification approaches to four datasets with experimental doublet annotations and find that our methods perform at least as well as the state of the art, at comparably little computational cost. We observe appreciable differences between methods and across datasets and that no approach dominates all others. In summary, scds presents a scalable, competitive approach that allows for doublet annotation of datasets with thousands of cells in a matter of seconds.

**Availability and implementation:**

scds is implemented as a Bioconductor R package (doi: 10.18129/B9.bioc.scds).

**Supplementary information:**

Supplementary data are available at *Bioinformatics* online.

## 1 Introduction

Single-cell RNA sequencing (scRNA-seq) technologies allow characterization of transcriptomes of individual cells and aid our understanding of tissue and cell-type heterogeneity. New insights, for instance in the context of development and/or disease (Li *et al.*, 2017; for a review see Potter, 2018; Segerstolpe *et al.*, 2016), have made them increasingly relevant across a diverse range of biomedical research fields. Specifically, approaches that enable the study of many thousands of cells simultaneously are making an impact, and the availability of user-friendly solutions (like the 10X Chromium platform, for example) has rendered scRNA-seq the assay of choice in numerous studies. However, use of scRNA-seq data is not without challenges, and careful data processing, quality control and analysis is essential (reviewed for instance in AlJanahi *et al.*, 2018; Kiselev *et al.*, 2019; Stegle *et al.*, 2015; Vallejos *et al.*, 2017). We focus on one key step of quality control that is the identification of so-called *doublets* (or *multiplets*). Doublets (or multiplets) arise in scRNA-seq data when two (or more) cells are mistakenly considered as a single cell, due for instance to being captured and processed in the same droplet on a micro-fluidics device. This type of error has the potential to severely confound interpretation of study results, especially in the context of cellular heterogeneity and identity, where they may appear as spurious novel cell types. However, despite rapid advances in the field, to our knowledge relatively few approaches exist that address the issue of doublet detection in scRNA-seq data. In the following, we provide a brief overview of existing experimental and computational approaches for doublet identification.

### 1.1 Experimental methods for doublet detection

For some approaches, doublet detection can be performed as a quality control step to ensure that only single cells are picked at capture sites (e.g. Proserpio *et al.*, 2016; Segerstolpe *et al.*, 2016). Alternatively, barcodes have been used together with mixtures of cells from different species to get estimates of doublet rates (e.g. Alles *et al.*, 2017; Klein *et al.*, 2015). In their work, Kang *et al.* (2018) present a multiplexing strategy that exploits genetic variation to detect doublets among mixtures of cells from different individuals. In another approach, Stoeckius *et al.* (2018) use oligonucleotide-tagged antibodies against cell surface proteins to uniquely label cells in a robust multiplexing strategy that allows for doublet detection. In a similar vein, Gehring *et al.* (2018) use chemical labeling for tagging cells from individual samples. Recently, McGinnis *et al.* (2019b) proposed a technique called MULTI-seq, which uses lipid-modified oligonucleotides to barcode individual cells. Thus, development of experimental approaches that improve doublet detection is a field of active research. However, experimental approaches typically face the limitation that they require specific technologies or experimental designs, which are often not readily available to researchers (for an overview of limitations of some of these approaches, see Wolock *et al.*, 2019). Therefore, it is at the stage of computational data analysis where approaches are needed to identify doublets.

### 1.2 Computational methods for doublet detection

There are few computational approaches that explicitly address the problem of distinguishing doublets from single cells using scRNA-seq expression data alone. Often, researchers rely on curated marker genes and expert knowledge to identify cells co-expressing markers of distinct cell types as putative doublets (e.g. Ibarra-Soria *et al.*, 2018; Rosenberg *et al.*, 2018; Wang *et al.*, 2016). Based on the assumption that doublets would have higher total RNA content, another approach is to use a measure for overall expression signal (total counts, for example) as a means for classifying cells as doublets (e.g. Bach *et al.*, 2017; Krentz *et al.*, 2018; Ziegenhain *et al.*, 2017). However, given that marker gene information and expert knowledge is not always available (and not always objective), and that doublets may not necessarily have high total counts, recently a number of computational doublet detection/annotation methods have been proposed that do not rely on markers or total counts alone (https://github.com/JonathanShor/DoubletDetection, DePasquale *et al.*, 2018; Lun *et al.*, 2016; McGinnis *et al.*, 2019a; Wolock *et al.*, 2019; Table 1). In the following, we briefly summarize each of them:

**Table 1.** Computational approaches for doublet annotation

Method	Language	Reference
scrublet	Python	Wolock *et al.* (2019)
dblDetection	Python	doi:/10.5281/zenodo.2658730
dblFinder	R	McGinnis *et al.* (2019a)
dblCells	R	Lun *et al.* (2016)
dblDecon	R	DePasquale *et al.* (2018)

scrublet: In their approach scrublet, Wolock *et al.* (2019) simulate artificial doublets from the original data coordinates of the normalized and filtered data in a reduced-dimensional representation obtained by principal component analysis (PCA). A *doublet score* is then created by considering the fraction of artificial doublets in the neighborhood of each barcode using k-nearest-neighbor (kNN) graph based on Euclidean distances. To determine the fraction of doublets in an experiment, a doublet score threshold is set visually by comparing the distributions of the doublet scores of original barcodes and artificial doublets. scrublet is available as a python module.

dblFinder: In a similar vein, DoubletFinder (McGinnis *et al.*, 2019a) also uses artificial doublets, and the fraction of artificial doublets in the neighborhood of each barcode, to calculate a metric (‘pANN’), akin to the doublet score discussed above. Artificial doublets are created by averaging raw counts of randomly paired barcodes, then the data are normalized, PCA performed and pANN scores computed. The authors provide a heuristic to automatically choose parameters (like the number of neighbors considered), and finally thresholding pANN based on the expected doublet rate [or based on an adjusted rate that accounts for homotypic doublets (doublets formed by cells of the same type)] yields final doublet annotations. dblFinder is available as an R package.

dblCells: In the vignette of their R package simpleSingleCell (Lun *et al.*, 2016) discuss two approaches, doubletClusters and doubletCells, implemented as part of the R package scran (Lun *et al.*, 2016). The first prescribes an approach to identify clusters of cells that have intermediate expression profiles to ‘parent’ clusters based on differentially expressed genes, library size and number of cells in a cluster (Bach *et al.*, 2017). Of relevance to us is the second approach doubletCells, whereby thousands of artificial doublets are generated by combining randomly chosen pairs of barcodes and projecting them into a reduced-dimensional space. A doublet score is formalized by assessing neighborhoods of simulated doublets and original barcodes.

dblDetection: This approach (http://doi.org/10.5281/zenodo.2658730) also relies on artificially generated doublets, but, in contrast to previous methods, performs cell clustering on the augmented dataset. Briefly, augmented data with artificial doublets is generated from one of two possible sampling schemes, projected into a lower-dimensional representation using PCA and then clustering is performed with phenograph (Levine *et al.*, 2015, https://github.com/JonathanShor/PhenoGraph). Next, hypergeometric *P*-values are assigned to clusters and their cells based on the number of artificial doublets they contain. This procedure (including artificial doublet generation) is performed multiple times, and then doublet calls and scores are derived from annotated *P*-values across runs/iterations. dblDetection is available as a python module.

dblDecon: Making use of an initial user-provided clustering, the method of DePasquale *et al.*, 2018, DoubletDecon, relies on deconvolution as implemented in the R package DeconRNASeq (Gong and Szustakowski, 2013) to identify doublets. First, distinct reference profiles are constructed from the initial clustering and then artificial doublets are generated and their deconvolution profiles are computed. Next, barcodes with deconvolution profiles closest (by Pearson correlation) to those of a synthetic doublet are initially predicted to be a doublet. Finally, to reduce penalizing cells with gene expression profiles possibly corresponding to transitional cell states, the authors implement a ‘rescue’ step whereby predicted doublets with unique gene expression patterns are re-labeled as single cells. dblDecon is available as an R package.

We note that most of these approaches are recent, based on similar strategies and to our knowledge have not been assessed together across multiple datasets in a systematic way. In the following, we present two new and complementary methods for computational doublet annotation: Co-expression based doublet scoring (cxds) and binary classification based doublet scoring (bcds). We show that they can accurately annotate doublets, and we perform a comparison of these approaches and the methods discussed above on four publicly available datasets with experimental doublet annotations (Table 2). We show that our methods perform well compared with existing approaches (at comparably little computational cost), and we demonstrate heterogeneity in results and performance of computational doublet annotation between different methods and across different datasets.

**Table 2.** Datasets with experimental doublet annotation

Dataset	Cells	Sparsity	# Genes	Reference
hgmm	12 820	79%	3068.5	10X Genomics
ch_pbmc	15 583	98%	321	Stoeckius *et al.* (2018)
ch_cell-lines	8191	92%	2086	Stoeckius *et al.* (2018)
demuxlet	14 619	97%	520	Kang *et al.* (2018)

*Notes*: The ‘# Genes’ column shows the median number (across cells) of expressed genes. The URL for the hgmm 10X Genomics dataset is https://support.10xgenomics.com/single-cell-gene-expression/datasets-2.1.0/hgmm_12k.

## 2 Materials and methods

### 2.1 Co-expression based doublet scoring

Co-expression based doublet scoring (cxds) is motivated by the assumption that heterotypic doublets (i.e. doublets comprised of cells from different cell types), co-express ‘marker’ genes that are not usually active in the same cell. In contrast to approaches that leverage expert knowledge and assess expression patterns of curated sets of marker genes manually, cxds uses the scRNA-seq data to first assess gene pairs and then derive an overall doublet score for each barcode (We use the terms ‘cell’ and ‘barcode’ interchangeably, and sometimes use ‘doublet cells’ to refer to barcodes coding for two or more cells, based on gene-gene co-expression).

Specifically, let $X\in R^{m\times n}$ be a genes × cells count matrix for *m* genes and *n* cells, and *B* its thresholded binarized version, where *B_ij_* denotes whether gene *i* is expressed in cell *j* (absence/presence). The row means of *B*, ${\left\{p_{k}\right\}}_{k=1}^{m}$, are the fraction of cells expressing each gene, and the symmetric matrix *BB^T^* contains for each gene pair the number of cells co-expressing the two genes. If we denote the matrix where the entries in *B* have been flipped by $\underline{B}$  $\left({\underline{B}}_{\mathrm{ij}}=1-B_{\mathrm{ij}}\right)$, then we can write the number of cells that express exactly one of two genes as $\left(B{\underline{B}}^{T}+\underline{B}B^{T}\right)$ and, assuming independence between genes, arrive at the following binomial model:

$${\left(B{\underline{B}}^{T}+\underline{B}B^{T}\right)}_{\mathrm{ij}}∼\text{Bin}(n,p_{i}(1-p_{j})+p_{j}(1-p_{i}))\;,$$

where (*ij*) now denotes a pair of genes. Let a ‘score matrix’ $S\in R^{m\times m}$ hold negative (upper tail) log *P*-values under the above model. Scores for gene pairs that co-express across cells ***less often*** than expected (given their marginal frequencies) are high, while scores for pairs that co-express normally (or more often than expected) are low. We now use *S* to derive cell-specific doublet scores by summing, for each cell, negative log *P*-values of co-expressed gene pairs, so that we get for cell *i* a doublet score cxds via:

(1) $$\text{cxds}(i)=\sum_{k}\sum_{j}B_{\mathrm{ki}}B_{\mathrm{ji}}S_{\mathrm{kj}}=\text{diag}{\left(B^{T}SB\right)}_{i}\;.$$

We then rank cells in the order of decreasing scores, with high scores denoting doublet cells/barcodes. We note that *B*, for UMI data, is typically sparse (often more than 95% zeros), so that the matrix products *BB^T^* and *B^T^SB* above are not prohibitive, even for tens of thousands of cells. On the contrary, our run times are comparable with the fastest current approaches (see Table 3 in the Section 3).

**Table 3.** Running time for doublet detection methods in seconds

# cells	1k	2k	4k	8k	12k
□ cxds	0	0	1	2	2
□ bcds7	1	2	4	7	8
○ scrublet	1	1	3	7	11
⋆ bcds	5	7	11	22	26
○ dblCells	19	41	106	210	340
○ dblFinder	58	93	207	384	627
○ dblDetection	56	102	228	484	774

*Notes*: The row for bcds7 denotes the time for bcds for a fixed number of training rounds (Section 2).

As mentioned above, a motivation for this score is a (simplified) concept of marker genes that are expressed in specific cell types only. Gene pairs containing marker genes for the same cell type will receive low scores (they are co-expressed more often than expected), while gene pairs with marker genes for different cell types would receive high scores (they are co-expressed less often than expected, because they do not co-express in non-doublet cells). In our cell-specific scores ${\left\{\text{cxds}(i)\right\}}_{i=1}^{n}$, we then aggregate information across gene pairs.

#### 2.1.1 Gene pair scoring

Because the doublet score cxds(·) in Equation (1) directly sums up contributions of individual gene pairs, we can rank pairs based on their cumulative impact on doublet prediction in the dataset at hand, weighted by the doublet score for each cell. For the ‘importance’ of a pair formed by genes *k* and *j* we define

(2) $$\text{imp}(k,j)=\sum_{i}\text{cxds}(i)B_{\mathrm{ki}}B_{\mathrm{ji}}S_{\mathrm{kj}}={\left((\mathrm{BD}B^{T})*S\right)}_{\mathrm{kj}},$$

where *D* is a diagonal matrix containing doublet scores and the asterisk denotes the element-wise product of matrices. This approach prioritizes gene pairs that substantially contribute to the annotation of cells with high doublet scores, and it can be used to study the pairs of genes that most drive doublet prediction. Further on, to prioritize gene pairs that drive doublet predictions in a particular cell we can omit the sum in Equation (2); or, to focus on a group of cells (forming a cluster, for instance), we can restrict the sum to group members.

#### 2.1.2 Implementation

We implemented cxds using the R programming language (R Core Team, 2018), and in practice add two heuristics: Given a count matrix *X* of an scRNA-seq experiment, we first binarize expression based on a threshold binThresh, such that *B* contains genes with more than binThresh counts. In all our studies, here we set binThresh to zero, but other values can be reasonable (Supplementary Table S1). Next, we focus on highly variable genes by ranking genes with respect to their Binomial variance (i.e. $np_{j}(1-p_{j})$ for gene *j*) then keeping only the ntop most variable ones. We choose ntop = 500 as default (Supplementary Table S2 shows that cxds results are largely robust with respect to different choices of the ntop parameter).

### 2.2 Binary classification based doublet scoring

Binary classification based doublet scoring (bcds) employs artificial doublets, similar to other strategies (see Section 1 for an overview). However, it does not rely on dimension reduction or nearest neighbor approaches to calculate a doublet score. Briefly, given a genes-by-cells matrix of expression counts we create artificial doublets by adding random pairs of columns. We then log-transform, normalize and select variable genes before using a binary classification algorithm to discriminate artificial doublets from original input data. Finally, for each input barcode we then take the estimated probability of belonging to the artificial doublet class as the doublet score we annotate.

#### 2.2.1 Implementation

We implemented bcds using the R programming language (R Core Team, 2018), with the following specifics. We simulate artificial doublets by randomly selecting pairs of cells and adding their counts, followed by mean-normalization of log-counts of all cells (artificial doublets and input cells) and thereby generate an augmented dataset containing input data and simulated doublets. We then train gradient boosted decision trees (Chen and Guestrin, 2016) using the xgboost R package (Chen *et al.*, 2019) with default parameters for artificial doublet classification. We employ two heuristics for establishing the number of training rounds: (i) We use 5-fold cross-validation approach in combination with the ‘one-standard-error-rule’ (Hastie *et al.*, 2001) to determine the number of rounds to train on the complete dataset. (ii) We set the number of training rounds to seven. In both cases, we stop training in case the misclassification error does not decrease for two consecutive rounds. All results reported in this manuscript use heuristic (i), except for Table 3, where we report running times; there we also report heuristic (ii), termed bcds7 (Supplementary Table S3 compares the performance of the two heuristics across datasets). We report the class probability for the artificial doublet class given by the model trained on the complete dataset as doublet scores. Also, like with cxds, we select ntop variable genes before simulating doublets and training the classifier. Here, we log-transform and mean-normalize count values before calculating the variance of each gene. The ntop most variable genes are then included for further analysis, and we choose ntop = 500 for all results reported.

### 2.3 Hybrid doublet scoring

We also combine both approaches, cxds and bcds, into a version generating annotations as follows. After running each method we simply normalize the scores to fall between zero and one (by subtracting the minimum and subsequently dividing by the maximum) before adding them. We denote these annotation scores as hybrid.

### 2.4 Data description, retrieval and processing

We evaluated our approach and compared performance with other methods on four publicly available datasets with experimentally annotated doublets. Table 2 lists the datasets, in the following we describe how we retrieved and processed each of them:

hg-mm: This dataset contains a 1:1 mixture of freshly frozen human HEK293T cells and mouse NIH3T3 cells. We downloaded data from the 10X genomics website (www.10xgenomics.com) and processed them as follows: Barcodes were filtered to include those with experimental doublet annotations. For genes, human-mouse 1:1 orthologs were identified using the Ensemble database (v95, Zerbino *et al.*, 2018) with the getLDS function provided by the biomaRt R software package (Durinck *et al.*, 2009), and corresponding counts were added. Removing features with no counts resulted in gene expression data of 14 437 orthologs across 12 820 barcodes.

ch_pbmc: This dataset contains peripheral blood mononuclear cells (PBMCs) from eight donors, with cells from each donor uniquely labeled using the cell hashing approach of Stoeckius *et al.* (2018). Data files were downloaded from Dropbox (https://www.dropbox.com/sh/c5gcjm35nglmvcv/AABGz9VO6gX9bVr5R2qahTZha? dl=0) and processed according to the vignette of the Seurat R package (Butler *et al.*, 2018) entitled ‘Demultiplexing with hashtag oligos (HTOs)’ (https://satijalab.org/seurat/hashing_vignette.html). This resulted in a gene expression matrix of 21 606 genes across 15 583 barcodes.

ch_cell-lines: This dataset contains a mixture of four human cell lines, HEK, K562, KG1 and THP1. Each cell line was labeled using the cell hashing approach of Stoeckius *et al.* (2018). Data files were downloaded from the same location as for ch_pbmc and processed according to the same vignette, resulting in a gene expression matrix with 25 241 genes across 8191 barcodes.

demuxlet: This dataset contains a uniform mixture of PBMCs from eight lupus patients, and doublets have been annotated based on genetic information using demuxlet (Kang *et al.*, 2018). Data files for gene expression counts were downloaded from GEO (GSM2560248) and doublet annotations were retrieved from the demuxlet github repository (https://github.com/statgen/demuxlet). This resulted in data comprising of expression counts for 17 662 genes across 14 619 barcodes.

We note that for all gene counts above, and for the sparsity calculations in Table 2, we included genes expressed with at least one count in one barcode.

### 2.5 Annotation of doublets with existing methods

We annotated doublets with five existing tools (Table 1), and in the following we describe how we applied each of them:

dblCells: Data were processed per the vignette of the R package simpleSingleCell (Lun *et al.*, 2016). Briefly, raw counts were normalized using size factors computed using scran (Lun *et al.*, 2016) with the igraph clustering method and a min.mean value of 0.1. Technical noise was removed using the denoisePCA function of scran with approximate singular value decomposition performed (approximate = TRUE). Finally, doublet scores were retrieved using the doubletCells function run with default options except again with approximate = TRUE to allow fast approximate PCA.

dblDecon: Raw counts were fully processed using Seurat (Butler *et al.*, 2018) [i.e. normalization, scaling with nUMI regressed out, finding variable genes, dimension reduction (with PCA) and clustering were performed]. Additionally, marker genes were calculated with default settings using the FindAllMarkers function and top 50 markers used. The Main_Doublet_Decon function was run with input files created using the Seurat_Pre_Process function and default settings except for species which was set to hsa, and using centroids as references for deconvolution (centroids = TRUE).

dblDetection: The python module (https://github.com/JonathanShor/DoubletDetection) was used in the R programming language using the reticulate package (https://github.com/rstudio/reticulate), and run with default parameters on the count data. For each cell, negative log *P*-values were averaged across runs/iterations to derive an aggregate doublet score for each cell.

dblFinder: Fully processed Seurat objects were created (Butler *et al.*, 2018), where normalization, scaling (with nUMI regressed out), finding variable genes (with arguments as per their github example code), dimension reduction (PCA and TSNE) and clustering were performed with dims.use = 10 and all other Seurat settings set to default. For dblFinder, the value for pK was selected following the best practices outlined on their github page (https://github.com/chris-mcginnis-ucsf/DoubletFinder), i.e. as the one with the maximum mean-variance normalized bimodality coefficient (BCmvn). The function DoubletFinder was run with the expected doublet rate of 7.5% assuming Poisson statistics, as per the example code on github (see URL above).

scrublet: The python module scrublet (Wolock *et al.*, 2019) was used in the R programming language using the reticulate package (https://github.com/rstudio/reticulate), and run with default parameters on raw count data. Doublet scores were used as reported by the software.

### 2.6 Data visualization and calculation of performance metrics

Low dimensional representation for visualization of data in our figures were calculated as follows: For each dataset, log counts were calculated and random projection PCA was performed on the 500 most variable genes using the rsvd R package (Erichson *et al.*, 2016); finally the first ten principal components were projected into two dimensions for visualization using the Rtsne package (Krijthe, 2015) with default parameters.

For performance evaluation, we calculated the area under the ROC curve using the pROC R package (Robin *et al.*, 2011), including partial areas under the ROC curve (pAUC) at 90%, 95% and 97.5% specificity. For the partial areas, the option partial.auc.correct was set to TRUE, such that the maximal pAUC is one and a pAUC of 0.5 is non-discriminant. Areas under the precision-recall curves (AUPRCs) were calculated using the PRROC package (Keilwagen *et al.*, 2014) and we report the smoothed area under the curve according to Davis and Goadrich (2006) by selecting the appropriate option. We used all cells present in each dataset (see above) to calculate performance metrics. We note that dblDetection would occasionally not score a small subset of cells (between 0 and 11), which we then excluded for this method’s metrics.

Running times for methods available as R packages were calculated in R, while python was used for python-based methods. The median (middle) value of three timings is reported. Methods were run on the same sub-samples of cells of the demu data, and four cores of an Intel(R) Xeon(R) E5-2667 v4 CPUs were made available for computing.

## 3 Results

**Fig. 1.**
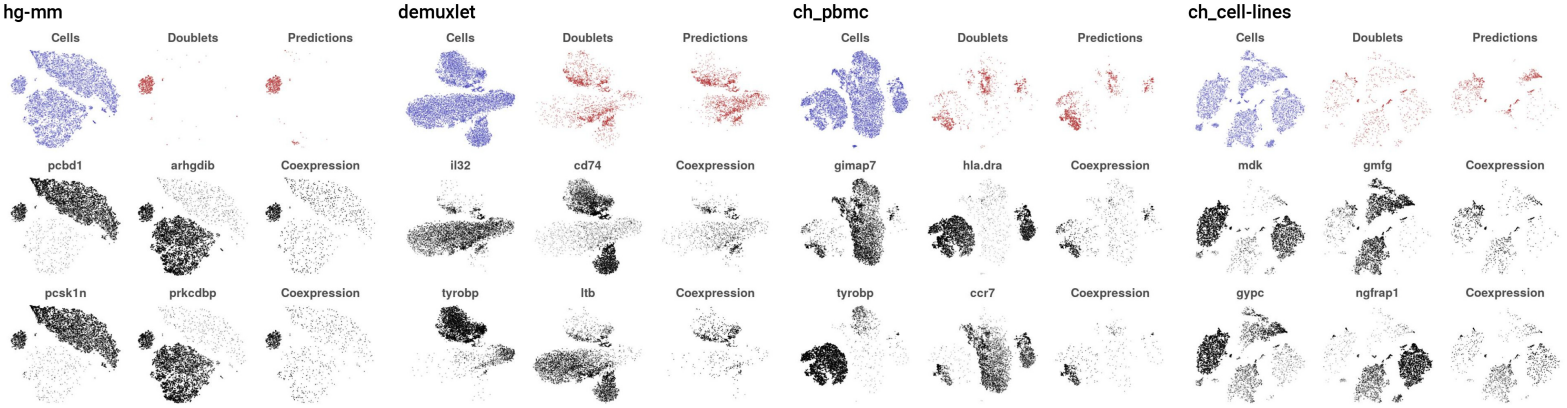
Gene pairs driving doublet prediction in cxds. For four datasets (panels) the first row shows all cells in (left), the annotated doublets (center) and cxds-predicted doublets (right). The following two rows depict the two gene pairs that contribute most to the cxds classifier (Section 2). For each pair (i.e. for each row), the left plot depicts the expression of one gene (presence/absence), the middle plot the expression of the other gene, while the right plot the average expression in cells that co-express both genes. We see that each gene in a pair is expressed in distinct groups of cells, and that their co-expression highlights annotated and predicted doublets

We report two computational methods for *in silico* doublet prediction: co-expression based doublet scoring (cxds) and binary classification based doublet scoring (bcds). Co-expression based doublet scoring identifies doublets from thresholded expression data essentially using a Binomial model (Section 2), based on the reasoning that marker genes for different cell types do not co-express in (non-doublet) barcodes. Pairs are scored exhaustively, and no prior knowledge about marker genes in a specific context is needed. Further on, doublet annotations for cells are interpretable in the sense that they are based on the co-expression of pairs of genes and cxds allows users to view gene pairs ordered with respect to their contribution to doublet predictions across a dataset (Section 2). Figure 1 shows the top two pairs driving doublet prediction for cxds across the four datasets (Table 2), illustrating how co-expression of genes in each pair identifies doublet cells.

Binary classification based doublet scoring, on the other hand, combines generation of artificial doublets from existing data with binary classification. Barcodes in the original data that are difficult to discriminate from artificial doublets receive high doublet scores using bcds. We use gradient boosted decision trees (Chen and Guestrin, 2016) to classify (Section 2), but in principle the approach is generic and other classification algorithms could be explored. In the following, we apply our methods to four datasets with experimental doublet annotations (Table 2), provide evidence that combining the cxds and bcds into a ‘hybrid’ score (Section 2) improves performance and compare our approaches and other computational methods for doublet annotation (Table 1).

### 3.1 Doublet scoring with scds accurately recapitulates experimental doublet annotations

We performed computational doublet annotation on four scRNA-seq datasets (Tables 2 and 4) using several current methods (Table 1), together with library size (libsize) and number of expressed genes (termed features); together both are referred to as ‘baseline’ methods from here onwards. Results for each dataset are presented in Supplementary Table S4 and performance averaged across datasets is shown in Table 4. Columns are performance metrics [the area under the receiver operating characteristic curve (ROC curve), the area under the precision-recall curve (PR curve) and partial areas under the ROC curve focusing on 90%, 95% and 97.5% specificity], while rows correspond to computational doublet annotation approaches. Rows are sorted with respect to their performance in terms of the area under the ROC curve (AUROC), with ties being broken by the performance in terms of the area under the PR curve (AUPRC). Baseline methods are marked with gray bullets, current methods with blue bullets and our proposed approaches with red bullets. We find that all the methods we propose (cxds, bcds and hybrid) perform well across datasets, consistently outperforming baseline approaches and at least one ranks in the top three best performing methods. The one exception is the ch_pbmc dataset (Supplementary Table S4), where annotating doublets based on the number of features achieves an area under the ROC curve of 79%. Our weakest-performing approach on this data, cxds, performs slightly worse (78%), but does much better in terms of area under the PR curve (AUPRC of 54% versus 45%, respectively). We also note that two other computational doublet annotation methods, dblCells and scrublet, perform worse than the number of features in terms of AUROC on this dataset. On average, our hybrid method does best of the three methods we propose, significantly outperforming baseline approaches on all four datasets.

**Table 4.** Performance of doublet annotation methods, averaged across datasets

	AUROC	pAUC900	pAUC950	pAUC975	AUPRC
○ dblCells	0.75	0.69	0.66	0.63	0.47
□ libsize	0.76	0.60	0.56	0.53	0.29
□ features	0.78	0.62	0.57	0.54	0.33
⋆ cxds	0.83	0.74	0.71	0.68	0.56
○ scrublet	0.83	0.75	0.72	0.69	0.60
⋆ bcds	0.84	0.75	0.70	0.63	0.56
⋆ hybrid	0.85	0.77	0.72	0.67	0.62
○ dblDetection	0.85	0.80	0.75	0.71	0.66
○ dblFinder	0.86	0.80	0.76	0.72	0.67

*Notes*: Squares mark baseline methods, circles mark current methods for doublet annotation and stars mark proposed methods. AUROC: Area under the ROC curve; AUPRC: area under the precision-recall curve; pAUC: partial area under the ROC curve (Section 2).

### 3.2 Coexpression-based doublet scoring highlights informative gene pairs

One of the features of cxds is its ability to provide gene pairs that drive doublet annotations of cells in a specific dataset (Section 2). As an illustration, Figure 1 shows the top two gene pairs driving cxds doublet annotation in each of the datasets we analyzed. For each dataset, the first row shows a two-dimensional representation of all cells (left), the subset of experimentally annotated doublets (middle) and the subset of doublets predicted by cxds (right). The next two rows depict gene pairs: Binarized expression (presence/absence) of one gene alone on the left, of the second gene in the pair in the middle and co-expression of both genes in the same cell on the right (also absence/presence). We see that cxds finds genes with complementary expression patterns that mark coherent groups of cells, and how co-expression of these genes contributes to doublet predictions. We note that while no clustering has been performed, genes included in high-scoring pairs by cxds often look like they mark different cell types, or combinations thereof, that may be present in the data.

### 3.3 Comparison of computational doublet scoring methods

We compared computational doublet annotation methods across four datasets; performance evaluation results averaged across datasets are shown in Table 4, dataset-specific results are shown in Supplementary Table S4, while Supplementary Figure S4 shows a resampling-based assessment of prediction robustness. In addition to aggregate performance measures reported in these tables and figures, Supplementary Figures S5 and S6 show a more fine-grained comparison between methods. In general, we find that computational doublet prediction performs best on the hg-mm dataset, followed by demuxlet and ch_pbmc, while it is most challenging for the ch_cell-lines data. Within each dataset there is appreciable spread of performance between the different methods, with most methods consistently outperforming baseline approaches. From Table 4 we see that, on average, dblFinder, dblDetection and our hybrid approach perform best. However, this order varies between datasets; for example on the demu dataset, bcds performs slightly better than hybrid (Supplementary Table S4). On the ch_pbmc dataset, the baseline classifier features does better than on other datasets, outperforming cxds, scrublet and dblCells. In general, library size and number of features identify doublets reasonably well (AUCs ≥ 78%, with the exception of the ch_cell-lines dataset), which motivated us to further explore the effect of library size on the performance of computational doublet annotation.

#### 3.3.1 Doublet annotation performance stratified by library size

**Fig. 2.**
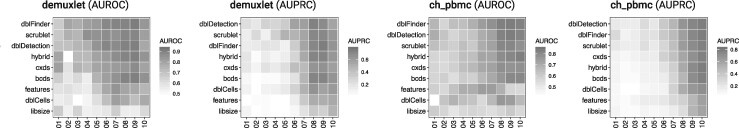
Performance of methods, stratified by library size. For two datasets, the first panel shows performance in terms of the area under the ROC curve (AUROC), while the second shows performance under the precision-recall curve (AUPRC), respectively. In each panel, the rows correspond to methods, and the columns to groups of cells in the same stratum of library sizes. The left-most column focuses on the 10% of cells with the lowest library size, the next column on the cells between the 10% and the 20% quantile and so on. In each panel methods are ranked by their average performance across quantile bins. See Supplementary Figure S1 for the remaining two datasets

For each dataset, we divided cells into equal-sized bins according to library size, so that the first bin contains cells with library sizes between the 0% and 10% quantile, the second bin cells between the 10% and 20% quantile, and so on. We then assessed annotation performance for all computational methods in each bin for each dataset separately. Results for demuxlet and ch_pbmc data are summarized in Figure 2, the remaining two datasets can be found in Supplementary Figure S1. For each dataset, the left panel depicts performance in terms of the area under the ROC curve (AUROC), the right panel in terms of the area under the PR curve (AUPRC). For each performance comparison, columns correspond to library size bins and rows to annotation methods.

We see that all approaches (baseline methods included) perform best on cells with high library size (quantile bins 5 and up), and that this trend is more pronounced for performance in terms of AUPRC, compared with AUROC. We also find that this trend applies broadly, with notable exceptions being: The hg-mm dataset, where most methods perform well in terms of AUROC across bins and dblDetection and cxds both also perform consistently across a wide range of bins in terms of AUPRC. The second exception is the demuxlet dataset, where hybrid and cxds perform suprisingly well in terms of AUROC for cells with small library sizes (first quantile bin).

#### 3.3.2 Comparison of doublet annotations between methods

**Fig. 3.**
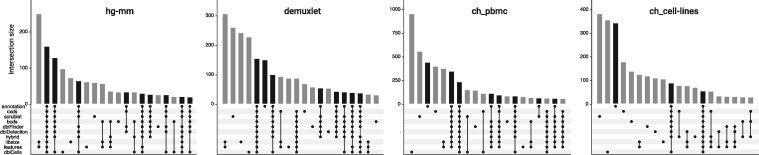
Comparison of doublet predictions. For the four datasets (panels), we show upset plots (Conway *et al.*, 2017) comparing doublet predictions for nine prediction methods (including baseline methods) with annotated doublet cells. Bars showing the size of intersections containing experimentally annotated doublets (termed ‘annotation’) are in black, bars showing intersections without experimentally annotated doublets are in gray. We show the 20 largest intersection sets. For demuxlet, ch_pbmc and ch_cell-lines the set of doublets that gets missed by all prediction methods (i.e. consistent false negatives) is ranked number six, three and three in terms of size, respectively

We also assessed similarities and differences between doublet predictions of each method. To do so, we determined the fraction of barcodes experimentally annotated as doublets and then compared the same number of doublet predictions for each method. Results are summarized in Figure 3, where we looked at overlapping and non-overlapping doublet annotations from different methods (and experimental annotations) in the form of upset plots (Conway *et al.*, 2017). Doublet annotations for each method (and experimental annotations) are considered as sets of barcodes, and this type of plot depicts set intersections, where the sets participating in each intersection are indicated by a ‘combination matrix’ at the bottom. Vertical bars indicate the number of cells in each such intersection class; bars colored in gray correspond to intersections containing only barcodes not experimentally annotated as doublet [i.e. false positives (FP)], whereas bars colored in black correspond to sets containing barcodes annotated as doublets [i.e. true positives (TP); the ‘annotation’ set is participating in these intersections]. The twenty largest intersection sets are shown for each dataset.

We find that in each dataset (except hg-mm) there is a substantial number of experimentally annotated doublet cells that none of the computational annotation approaches recovers (black bars corresponding to the ‘annotation only’ intersection). The cxds, scrublet and dblCells methods often have a fairly large amount of FP predictions that are unique to the respective methods, as do libsize and/or features. While we note these differences, we also see that TP predictions are typically shared by many methods. In fact, with the exception of the scrublet-specific TP predictions in the ch_pbmc data, all TP intersections have consistent predictions from at least four methods. That is, we observe better agreement between methods in terms of TP predictions as compared with FP predictions.

Further on, we compared the library size of cells, stratified by their annotation classes [TP, true negative (TN), FP and false negative (FN) predictions] for each method and dataset. Results are summarized in Supplementary Figure S2. We see that for some methods (cxds, bcds, hybrid, dblDetection, dblFinder) FP predictions tend to have higher library size compared with FN predictions, often comparable to TP predictions (similar to what McGinnis *et al.*, 2019a observe for dblFinder). We also find that this trend can vary for the same method between datasets (e.g. cxds has this trend in all datasets except ch_pbmc, and dblFinder has it in all datasets except hg-mm). This trend is markedly less pronounced in scrublet and dblCells.

Finally, the ch_cell-lines dataset contains experimental annotation about whether a doublet is homotypic (from the same cell line) versus heterotypic. We used this to quantify the enrichment of TP predictions for heterotypic doublets across methods. Results are summarized in Supplementary Table S5. We see that all methods (except dblCells) are significantly enriched for heterotypic doublets, with enrichment being most extreme for scrublet and dblDetection (odds ratio > 6), present for the remaining methods (odds ratios between three and four) and absent for the baseline methods (odds ratios of 1.2 and 0.98 for features and libsize, respectively). Next, we visually compared doublet annotations across methods and datasets.

#### 3.3.3 Visual comparison of annotated doublets

**Fig. 4.**
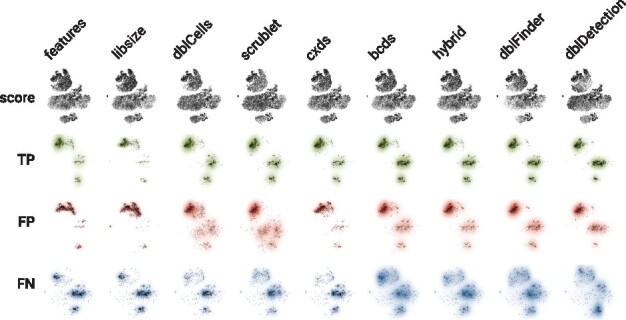
Visual comparison of doublet predictions for the demuxlet dataset. For nine computational doublet annotation methods (columns) cells are shown in a two-dimensional tSNE projection. The first row depicts all cells, shaded by the rank of the respective doublet prediction score. The second, third and fourth rows show true positive (TP, green), false positive (FP, red) and false negative (FN, blue) predictions. Shading reflects the relative density in each row, cells are shown in black

We visually compared doublet predictions. Figure 4 depicts that for the demuxlet data, whereas Supplementary Figure S3 holds results for all four datasets. In Figure 4, columns correspond to computational annotation methods and the first row shows doublet scores with darker colors representing higher scores (i.e. more doublet-like barcodes); the second, third and fourth rows show TP, FP and FN predictions, respectively. The relative density for each type of prediction is indicated in color (TP: green, FP: red, FN: blue). As before, we choose method-specific cutoffs such that the number of predicted doublets matches the number of experimentally annotated doublets.

For Figure 4 (the demuxlet data), we observe clearly visible differences in terms of TP, FP and FN density for all methods. We see FN predictions are more concentrated in the first five columns and more heterogenous in the other methods. This coincides with higher FP concentration for the baseline methods and cxds, but not for dblCells and scrublet. For the hg-mm data, where computational annotations are mostly correct (Supplementary Table S4), we see that TP and FN predictions are highly concentrated (Supplementary Figure S3). However, we can still make out interesting differences between the methods in terms of where their FP predictions fall. dblDetection, features and libsize have FP predictions in similar areas for one type of cells pretty much exclusively, scrublet and dblFinder predict false positives more predominantly in the other cell type, while hybrid and bcds have FP predictions in both types of cells. For the ch_cell-lines dataset, TP and FN predictions appear similar amongst non-baseline methods, while FP predictions appear distinct. For example, for dblDetection the FP density is highest in two of the four cell types (somewhat similar to the baseline methods), while for other methods FP predictions appear more broadly distributed. For the ch_pbmc dataset, we observe the biggest difference in terms of FP density between the two baseline methods and the rest.

Overall, we observe appreciable variability between doublet prediction methods, including the top three performers in Table 4, dblFinder, hybrid and dblDetection. This may suggest that none of the methods are close to optimal, and that an approach combining their respective strengths might further improve doublet annotation.

#### 3.3.4 Running time comparison

We measured running times of the different methods we compare, and Table 3 summarizes the results. We find that cxds, bcds_7 (where we do not perform cross validation, see Section 2) and bcds are able to annotate >10k cells in tens of seconds or on the order of a minute, while other methods take significantly longer. There is a distinct gap between ‘fast methods’, comprising the tools we propose and scrublet and the rest. We note that computational doublet annotation can be performed for each chip/batch separately, and therefore we did not assess larger numbers of barcodes.

#### 3.3.5 Comparison with dblDecon

dblDecon (DePasquale *et al.*, 2018) does not provide a doublet score, and therefore we could not include it in the previous analyses. To be able to still include it in our study, we applied it to all four datasets and generated doublet predictions. For the hg-mm dataset the method failed to run through, and therefore we excluded it from this analysis. For the three remaining datasets and other tools in the comparison, we then generated the same number of annotated doublets as dblDecon by choosing appropriate score thresholds. Surprisingly, we find that dblDecon does not perform well in this comparison, even though it determined the number of positive doublet calls for all methods (Supplementary Table S6). We also see a wide range of precision and sensitivity values across methods, while specificities are high due to the large amount of true negatives in all datasets.

## 4 Discussion

We have introduced single cell doublet scoring, scds, encompassing three methods (cxds, bcds and hybrid) for the *in silico* annotation of doublets in scRNA-seq data. We applied them to four datasets with experimental doublet annotations, and they all outperform baseline approaches. cxds is based on co-expression of gene pairs, and it is quite different from current approaches, because it does not utilize artificially generated doublets and it works on a binarized absence/presence version of the RNA expression data. It features fast running times and provides users the opportunity to investigate pairs of genes driving doublet predictions (Sections 2 and 3). Binary classification based doublet scoring, bcds, is more similar to established methods and utilizes artificially generated doublets. However, in contrast to other tools (see Section 1 for short descriptions), it does not make use of dimension reduction techniques, nor does it employ nearest neighbors for doublet scoring. Finally, hybrid is a combination of cxds and bcds that performs better than either method alone. In summary, our approaches are complementary to existing tools and work well for annotating doublets in scRNA-seq data.

We note that we do not estimate the number of doublets in a dataset, but rather score cells/barcodes and rank them from most doublet-like to least doublet-like. Therefore, our annotations are most useful when an estimate about the expected doublet rate is available (for instance, 10X Genomics provides them in their *‘User Guide for Chromium Single Cell 3’ Reagent Kits’*, based on the number of cells loaded on a chip), or when researchers wish to include a doublet score as only one of many factors in their decision about which cells may be excluded prior to downstream analyses. Our approaches share some conceptual limitations with other methods, which have been discussed in the literature (e.g. Wolock *et al.*, 2019). Specifically, successful doublet identifications require that doublets are rare, that mixtures of more than two cells are even more rare and that single cell instances of cell types in doublets are present in the data at appreciable frequency. Further on, our approaches are more sensitive towards identifying heterotypic doublets as compared with doublets comprised of two cells of the same type (also see Supplementary Table S5).

We also compared our methods and four existing tools that provide doublet scores using four datasets, and we find appreciable heterogeneity between computational doublet annotation methods. No tool consistently outperforms all others, and performance varies between datasets. Our tools perform well, especially when running time is a consideration. Averaged across datasets, dblFinder, dblDetection and hybrid are the top performing methods (Table 4). Investigating doublet predictions of each method in more detail, we find that: (i) for most datasets there is a sizable fraction of experimentally annotated doublets that is consistently missed by all methods, (ii) many correctly annotated doublets share the consensus of most methods and (iii) methods differ mostly in terms of their false positive annotations and these tend to be method-specific (i.e. typically not shared between methods). This implies that while methods differ in their doublet annotations (appreciable variability in terms of false positives), no method is yet able to recover a sizable fraction of annotated doublets (false negatives shared by all approaches). Therefore, we believe there is room to further improve computational doublet annotation. Specifically, we note that for bcds we used default parameters and did not really engage in parameter tuning, which could in principle lead to substantial improvements. The reason is that with only four doublet datasets available, we believe that there is some danger of inadvertent ‘information leak’ and therefore optimizing parameters may lead to over-fitting. But this concern will decrease as more (and more diverse) data with experimental doublet annotations become available.

We note that results of our method comparisons necessarily depend on the datasets we used, and how they were processed. We attempted to minimize processing steps as much as possible, and we did not filter cells in addition to the original publications. We used published/provided experimental annotations, and for cell hashing (Stoeckius *et al.*, 2018) we followed the annotation strategy prescribed by the authors (Section 2). However, we are cognizant that alternative data processing strategies are equally reasonable and may have the potential to impact results. Further on, many analysis steps include random sampling in some way, thereby inducing a certain amount of stochasticity. Therefore, we have made the code for our analyses available (https://github.com/kostkalab/scds_manuscript) and provide a docker container (https://hub.docker.com/r/kostkalab/scds) for other researchers. Finally, in our study we used experimentally annotated doublets as gold standard ignoring shortcomings of the respective experimental approaches, for example, that some are not able to identify identically barcoded doublets (McGinnis *et al.*, 2019a). However, in the absence of better experimental data, we feel there are little alternatives to this approach.

In summary, *in silico* doublet annotation enriches single-cell RNA sequencing data and can guard against over interpretation of results. From our comparison, we find that current approaches (including ours) are able to annotate doublets more accurately than baseline methods, but also that there appears to be room for improvement as more datasets with experimental annotations become available. We introduced new light-weight methods for computational doublet annotation, which perform well in comparison to the status quo. They all feature comparably short running times, and co-expression based doublet scoring produces biologically interpretable results. Therefore, we provide researchers with new and useful tools to study and increase the value of their single-cell RNA sequencing data.

## Funding

This work was supported by the National Institute of General Medical Sciences of the National Institutes of Health under award number R01GM115836, and by the University of Pittsburgh School of Medicine.

*Conflict of Interest*: none declared.

## Supplementary Material

btz698_Supplementary_Data
