# Supplementary material for: scds: computational annotation of doublets in single-cell RNA sequencing data
Source: Bioinformatics. 2019 Sep 10;36(4):1150–8. doi: 10.1093/bioinformatics/btz698 (PMC7703774; doi:10.1093/bioinformatics/btz698)
Supplement: btz698_Supplementary_Data [file btz698_supplementary_data.pdf]

scds: Computational Annotation of Doublets in Single-Cell RNA Sequencing Data — Supplemental Material

Abha Bais[1,3] and Dennis Kostka[1,2,3]

[1] Department of Developmental Biology
[2] Department of Computational and Systems Biology and Center for Evolutionary Biology and Medicine
[3] University of Pittsburgh School of Medicine

Supplemental Tables

data	binThresh	AUROC	AUPRC	pAUC950	pAUC990	rec05	prec05
**chcl**							
chcl	4	0.667	0.320	0.583	0.556	0.207	0.449
chcl	5	0.666	0.317	0.582	0.552	0.206	0.446
chcl	3	0.662	0.312	0.581	0.557	0.200	0.434
chcl	1	0.655	0.294	0.577	0.547	0.204	0.441
chcl	0	0.652	0.265	0.568	0.528	0.198	0.429
chcl	2	0.650	0.296	0.575	0.554	0.191	0.415
**chpb**							
chpb	0	0.778	0.539	0.653	0.559	0.244	0.796
chpb	1	0.714	0.413	0.592	0.545	0.204	0.665
chpb	5	0.714	0.376	0.576	0.527	0.189	0.618
chpb	2	0.712	0.406	0.590	0.542	0.205	0.669
chpb	3	0.711	0.399	0.590	0.538	0.206	0.671
chpb	4	0.711	0.384	0.583	0.534	0.196	0.640
**demu**							
demu	0	0.886	0.493	0.634	0.528	0.278	0.595
demu	1	0.806	0.345	0.591	0.529	0.240	0.514
demu	2	0.783	0.316	0.579	0.526	0.216	0.462
demu	3	0.780	0.310	0.577	0.528	0.212	0.454
demu	4	0.776	0.303	0.574	0.524	0.213	0.457
demu	5	0.773	0.300	0.572	0.523	0.205	0.439
**hgmm**							
hgmm	1	0.995	0.979	0.984	0.968	0.860	0.994
hgmm	0	0.994	0.970	0.978	0.958	0.858	0.992
hgmm	2	0.994	0.970	0.977	0.957	0.856	0.989
hgmm	3	0.992	0.961	0.970	0.945	0.845	0.977
hgmm	4	0.992	0.955	0.966	0.938	0.835	0.966
hgmm	5	0.991	0.952	0.963	0.934	0.830	0.959

Supplemental Table 1: ***Effect of the `binThresh` parameter on cxds performance.*** For each data set we show results for different values of the `binThresh` parameter for the `cxds` method. We find that for `chpb` and `demu`, zero is the preferred value; `hgmm` is relatively robust with respect to changes in this parameter, while for `chcl` higher values, like four or five, have higher AUPRC than lower values. Again, the effect on AUROC is less pronounced. AUROC: Area under the ROC curve, pAUC`***`: Partial area under the ROC curve at indicated specificity, AUPRC: Area under the precision-recall curve (average precision), prec05: precision considering the 5% highest scores, rec05: recall considering the 5% highest scores.

data	ntop	AUROC	AUPRC	pAUC950	pAUC990	rec05	prec05
**chcl**							
chcl	900	0.667	0.346	0.602	0.572	0.240	0.520
chcl	1000	0.667	0.346	0.601	0.572	0.240	0.520
chcl	800	0.664	0.341	0.599	0.569	0.236	0.512
chcl	700	0.663	0.330	0.593	0.564	0.225	0.488
chcl	600	0.661	0.321	0.589	0.563	0.214	0.463
chcl	500	0.652	0.265	0.568	0.528	0.198	0.429
chcl	400	0.650	0.262	0.567	0.527	0.197	0.427
chcl	300	0.649	0.258	0.567	0.525	0.197	0.427
chcl	200	0.638	0.217	0.544	0.513	0.156	0.339
**chpb**							
chpb	900	0.782	0.544	0.656	0.558	0.245	0.799
chpb	1000	0.782	0.544	0.656	0.558	0.244	0.795
chpb	800	0.781	0.544	0.656	0.559	0.244	0.795
chpb	700	0.781	0.543	0.655	0.559	0.243	0.794
chpb	600	0.779	0.541	0.654	0.558	0.244	0.797
chpb	500	0.778	0.539	0.653	0.559	0.244	0.796
chpb	200	0.776	0.536	0.646	0.559	0.240	0.783
chpb	300	0.775	0.538	0.652	0.560	0.244	0.795
chpb	400	0.774	0.533	0.650	0.558	0.241	0.787
**demu**							
demu	700	0.888	0.496	0.635	0.528	0.277	0.592
demu	1000	0.888	0.495	0.632	0.529	0.274	0.587
demu	900	0.887	0.492	0.631	0.529	0.276	0.591
demu	500	0.886	0.493	0.634	0.528	0.278	0.595
demu	600	0.886	0.492	0.633	0.528	0.278	0.595
demu	800	0.886	0.490	0.632	0.529	0.277	0.592
demu	400	0.882	0.488	0.632	0.528	0.278	0.595
demu	300	0.881	0.497	0.640	0.529	0.281	0.602
demu	200	0.868	0.474	0.635	0.527	0.279	0.598
**hgmm**							
hgmm	1000	0.995	0.974	0.980	0.962	0.858	0.992
hgmm	700	0.994	0.973	0.979	0.963	0.860	0.994
hgmm	800	0.994	0.973	0.980	0.963	0.861	0.995
hgmm	900	0.994	0.973	0.980	0.962	0.861	0.995
hgmm	500	0.994	0.970	0.978	0.958	0.858	0.992
hgmm	600	0.993	0.970	0.977	0.960	0.860	0.994
hgmm	400	0.992	0.966	0.975	0.952	0.852	0.984
hgmm	300	0.992	0.955	0.966	0.937	0.839	0.970
hgmm	200	0.991	0.944	0.959	0.920	0.821	0.949

Supplemental Table 2: ***Effect of the `ntop` parameter on `cxds` performance.*** For each data set we show results for different values of the `ntop` parameter for the `cxds` method. We find that varying `ntop` within a reasonable range has little effect on performance. While a value of 500 (our default) is not optimal, changes typically have small results on performance. An exception is the `chcl` data set, where large values for ntop (900, 1000) increase the area under the precision-recall curve (AUPRC) from 27% to 35% (AUROC increases from 65% to 67%). All other parameters for `cxds` were at default values. AUROC: Area under the ROC curve, pAUC`***`: Partial area under the ROC curve at indicated specificity, AUPRC: Area under the precision-recall pdfcurve (average precision), prec05: precision considering the 5% highest scores, rec05: recall considering the 5% highest scores.

	AUC	pAUC900	pAUC950	pAUC975	AUPRC
**ch_cell-lines**					
bcds	0.69	0.66	0.65	0.63	0.43
cxds	0.65	0.59	0.57	0.55	0.26
bcds7	0.69	0.65	0.63	0.61	0.40
**ch_pbmc**					
bcds	0.81	0.71	0.66	0.60	0.58
cxds	0.78	0.69	0.65	0.61	0.54
bcds7	0.81	0.71	0.66	0.61	0.58
**demuxlet**					
bcds	0.91	0.79	0.70	0.61	0.59
cxds	0.89	0.71	0.63	0.57	0.49
bcds7	0.90	0.78	0.70	0.62	0.60
**hg-mm**					
bcds	0.96	0.85	0.77	0.69	0.62
cxds	0.99	0.98	0.98	0.97	0.97
bcds7	0.97	0.91	0.86	0.78	0.74

Supplemental Table 3: ***Heuristics for `bcds`.*** This table shows the performance of two different heuristics to determine the number of training rounds for `bcds` (see **Methods** Section in the main text). The rows labeled `bcds` use cross validation, while the rows labeled `bcds7` fix the number of training rounds to seven. AUROC: Area under the ROC curve, pAUC`***`: Partial area under the ROC curve at indicated specificity, AUPRC: Area under the precision-recall-curve (average precision).

	AUROC	pAUC900	pAUC950	pAUC975	pAUC990	AUPRC	TP
**chcl: (889 annotated doublets)**							
libsize	0.60	0.54	0.53	0.52	0.51	0.17	181
features	0.60	0.55	0.54	0.53	0.51	0.19	195
dblCells	0.64	0.62	0.61	0.60	0.59	0.37	296
cxds	0.65	0.59	0.57	0.55	0.53	0.26	261
scrublet	0.68	0.64	0.64	0.63	0.61	0.41	320
dblDetection	0.68	0.66	0.66	0.65	0.65	0.45	339
bcds	0.69	0.66	0.65	0.63	0.60	0.43	354
dblFinder	0.69	0.66	0.65	0.65	0.64	0.45	351
hybrid	0.70	0.64	0.62	0.60	0.59	0.40	333
**chpb: (2545 annotated doublets)**							
dblCells	0.63	0.57	0.56	0.54	0.52	0.31	807
libsize	0.78	0.63	0.57	0.54	0.51	0.44	1282
scrublet	0.78	0.67	0.63	0.59	0.55	0.52	1287
cxds	0.78	0.69	0.65	0.61	0.56	0.54	1309
features	0.79	0.62	0.57	0.54	0.52	0.45	1248
bcds	0.81	0.71	0.66	0.60	0.56	0.58	1503
hybrid	0.82	0.73	0.68	0.62	0.56	0.61	1544
dblDetection	0.83	0.75	0.69	0.62	0.56	0.63	1655
dblFinder	0.84	0.74	0.68	0.62	0.56	0.64	1623
**demu: (1565 annotated doublets)**							
dblCells	0.79	0.70	0.65	0.60	0.55	0.46	789
libsize	0.81	0.58	0.55	0.53	0.52	0.30	461
features	0.85	0.62	0.57	0.55	0.52	0.37	580
scrublet	0.87	0.74	0.68	0.62	0.55	0.54	886
cxds	0.89	0.71	0.63	0.57	0.53	0.49	851
dblDetection	0.90	0.79	0.69	0.59	0.53	0.58	1039
hybrid	0.91	0.78	0.68	0.59	0.53	0.58	1018
bcds	0.91	0.79	0.70	0.61	0.54	0.59	1003
dblFinder	0.92	0.79	0.70	0.63	0.56	0.62	996
**hgmm: (741 annotated doublets)**							
libsize	0.87	0.66	0.59	0.54	0.51	0.26	242
features	0.89	0.68	0.60	0.55	0.52	0.30	261
dblCells	0.93	0.88	0.84	0.79	0.71	0.73	530
bcds	0.96	0.85	0.77	0.69	0.61	0.62	453
hybrid	0.98	0.93	0.89	0.86	0.84	0.87	558
scrublet	0.99	0.96	0.94	0.91	0.85	0.91	613
cxds	0.99	0.98	0.98	0.97	0.96	0.97	683
dblDetection	0.99	0.98	0.97	0.97	0.97	0.97	705
dblFinder	1.00	0.99	0.99	0.99	0.97	0.98	706

Supplemental Table 4: ***Performance of doublet prediction methods across data sets.*** AUROC: Area under the ROC curve, pAUC***: Partial area under the ROC curve at indicated specificity, AUPRC: Area under the precision-recall-curve (average precision).

	scrublet	dblDetection	dblCells	dblFinder	hybrid	bcds	cxds	features	libsize
odds_ratio	6.90	6.01	3.97	3.32	3.11	3.00	3.04	1.20	0.98
p_value	4.01e-19	2.86e-18	2.29e-11	2.20e-10	2.45e-09	3.68e-09	1.06e-07	4.24e-01	9.18e-01

Supplemental Table 5: ***Enrichment of heterotypic doublets in true positive annotations.*** Columns code for different annotation methods. OR = odds ratio. P-values are via a Fisher exact test.

	ch_cell-lines				ch_pbmc				demu			
	TP	sen	spe	pre	TP	sen	spe	pre	TP	sen	spe	pre
libsize	93	0.10	0.96	0.26	460	0.18	0.97	0.56	504	0.32	0.91	0.29
features	112	0.13	0.97	0.31	471	0.19	0.97	0.57	645	0.41	0.92	0.37
dblDecon	199	0.22	0.98	0.55	396	0.16	0.97	0.48	539	0.34	0.91	0.31
dblCells	211	0.24	0.98	0.58	409	0.16	0.97	0.50	836	0.53	0.93	0.48
cxds	162	0.18	0.97	0.45	651	0.26	0.99	0.79	915	0.58	0.94	0.53
scrublet	253	0.28	0.98	0.70	619	0.24	0.98	0.75	932	0.60	0.94	0.54
hybrid	220	0.25	0.98	0.61	657	0.26	0.99	0.80	1102	0.70	0.95	0.63
bcds	260	0.29	0.99	0.72	642	0.25	0.99	0.78	1084	0.69	0.95	0.62
dblFinder	273	0.31	0.99	0.75	667	0.26	0.99	0.81	1077	0.69	0.95	0.62
dblDetection	284	0.32	0.99	0.78	662	0.26	0.99	0.81	1106	0.71	0.95	0.63

Supplemental Table 6: ***Comparison of doublet annotation methods with `dblDecon`.*** TP: True Positives, sen: sensitivity, spe: specificity, pre: precision. Rows are ordered by average precision.

Supplemental Figures

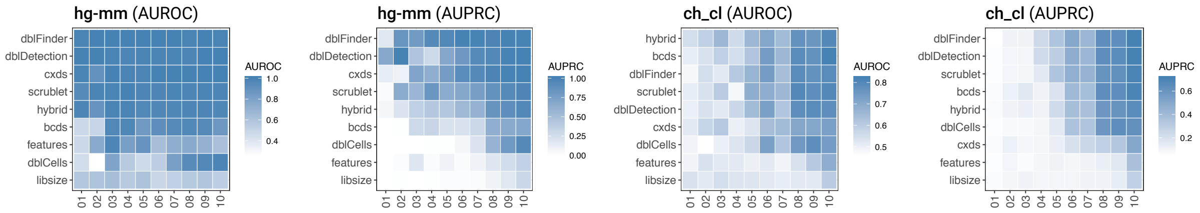

Supplemental Figure 1: ***Performance of methods, stratified by library size.*** For two data sets, the first panel shows performance in terms of the area under the ROC curve (AUROC), while the shows performance under the precision-recall curve (AUPRC), respectively. In each panel the rows correspond to methods, and the columns to groups of cells in the same stratum of library sizes. The left-most column focuses on the 10% of cells with the lowest library size, the next column on the cells between the 10% and the 20% quantile, and so on. In each panel methods are ranked by their average performance across quantile bins.

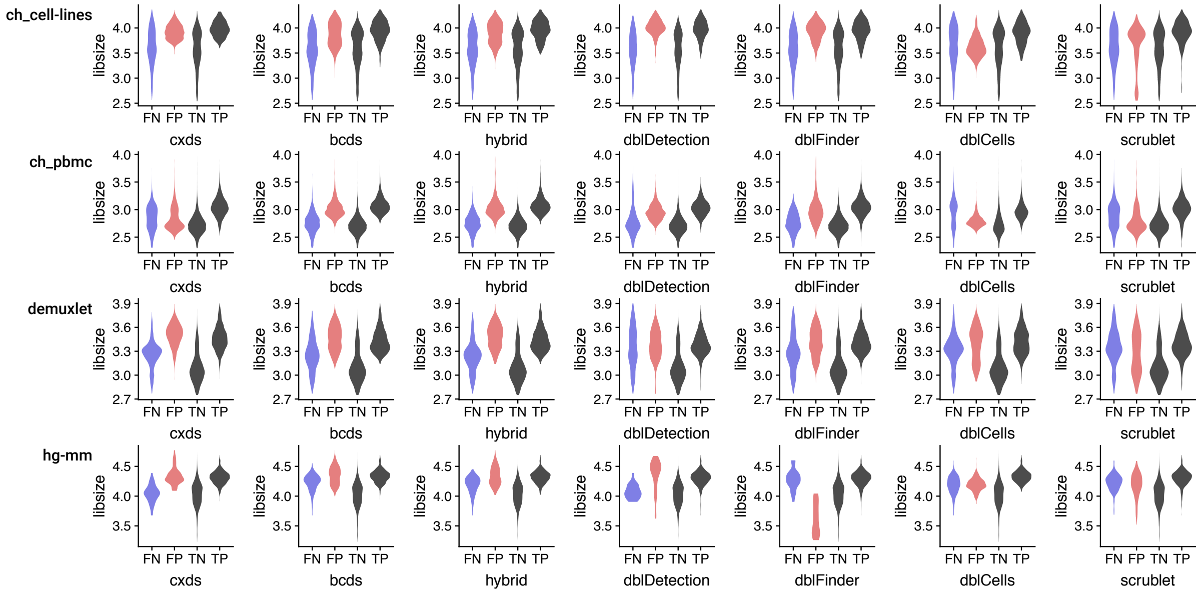

Supplemental Figure 2: ***Library size of doublet annotations, stratified by prediction type.*** For each data set (rows) and each method (columns) violin plots of library sizes are shown for false negative predictions (FN, blue), false positive predictions (FP, red) and true negative and true positive predictions (TN and TP, both black). For most (but not all) method/dataset combinations library size in FP predictions tends to be higher compared with FN predictions.

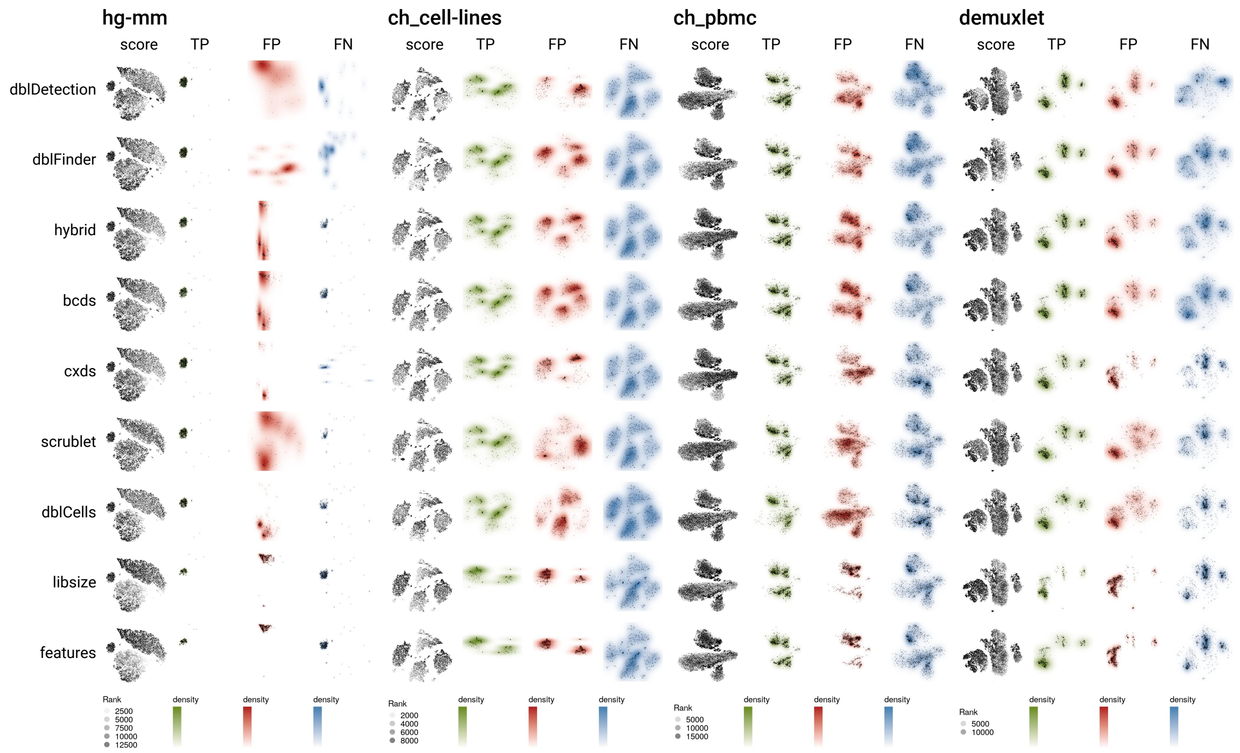

Supplemental Figure 3: ***Visual comparison of doublet predictions.*** For each data set (major columns), we show four panels (minor columns) for the nine methods we compared (rows). The first (left-most) panel depicts all cells, shaded by the rank of the respective doublet prediction score for a method. The second, third and fourth panels show true positive (TP, green), false positive (FP, red) and false negative (FN, blue) predictions for a method, respectively. Shading reflects the relative density, cells are shown in black.

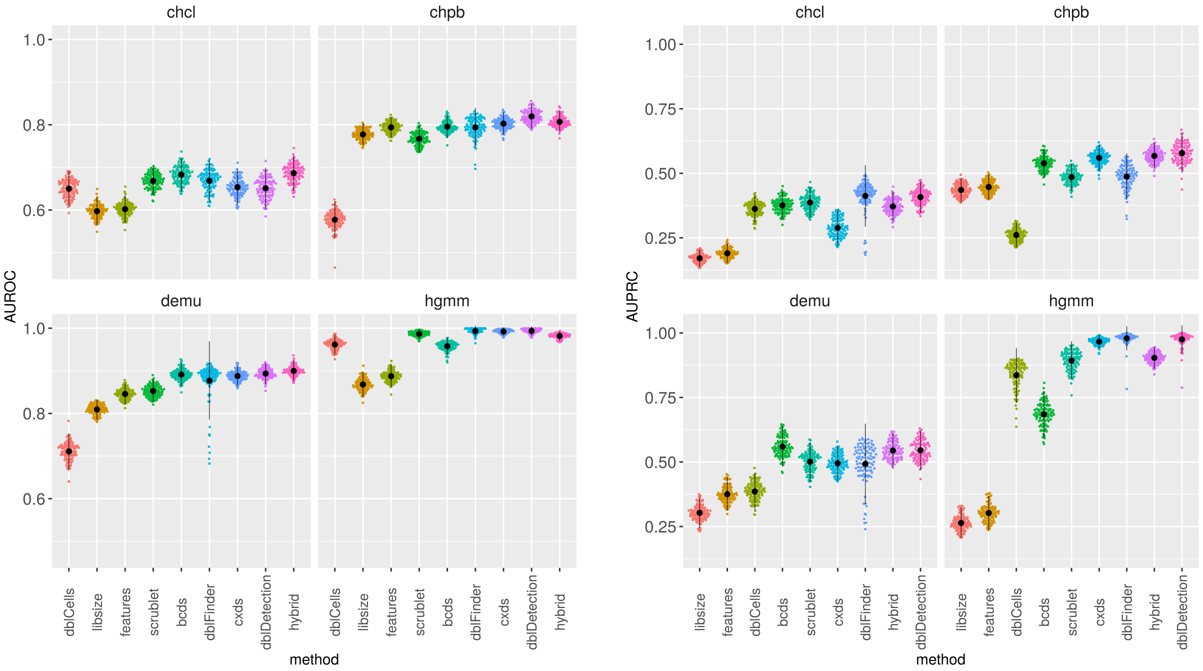

Supplemental Figure 4: ***Robustness of computational doublet predictions.*** We repeatedly (100 times) randomly sampled 2,000 cells from each of the four data sets and ran each doublet prediction method on the resulting subsets. On the left, performance is shown in terms of AUROC (area under the ROC curve), and on the right in terms of AUPRC (area under the precision-recall curve). We see that while performance is generally in line with expectations from analyses of the complete data sets, there is appreciable variability in performance for fixed method/dataset combinations between resampling runs.

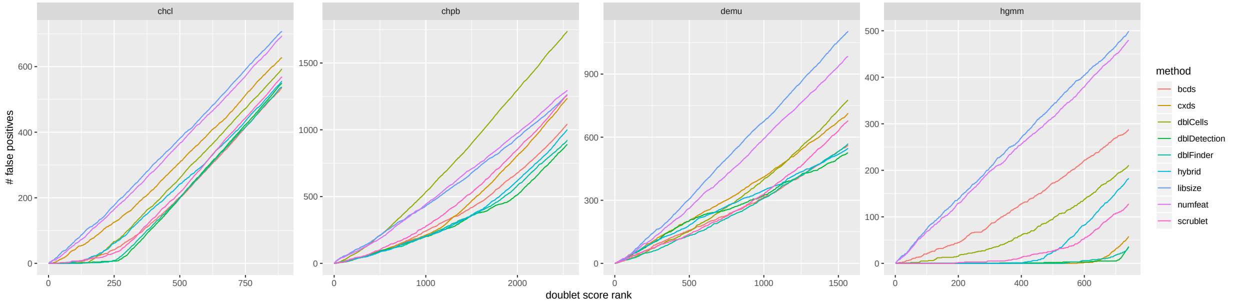

Supplemental Figure 5: ***Number of false positive predictions for computational doublet annotation methods.*** For each data set and each method, barcodes are ranked by their doublet annotation scores (x-axis, ranks). The y-axis shows the (cumulative) number of false positive doublet annotations.

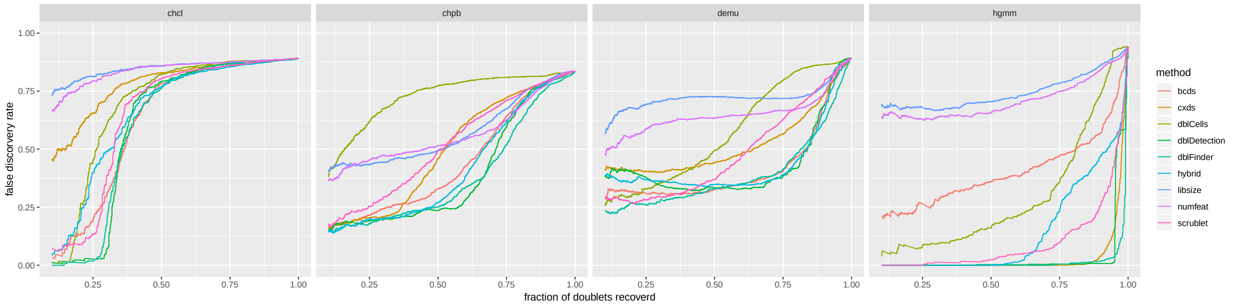

Supplemental Figure 6: ***False discovery rates for computational doublet annotation methods.*** For each data set and each method, barcodes are ranked by their doublet annotation scores (x-axis, scaled to reflect the corresponding fraction of experimental doublets recovered). The y-axis shows the false discovery rate.
